# Different ages of gut microbiota diversity and metabolic pattern in rhesus macaques

**DOI:** 10.3389/fmicb.2026.1747272

**Published:** 2026-07-28

**Authors:** Kaixuan Lv, Hongju Yang, Shuchao Ren, Yanchao Duan, Shuai Qiu, Tianzhuang Huang, Dan Wang, Hong Wang, Yaping Yan

**Affiliations:** 1 State Key Laboratory of Primate Biomedical Research, Institute of Primate Translational Medicine, Kunming University of Science and Technology, Kunming, Yunnan, China; 2 Southwest United Graduate School, Kunming, Yunnan, China; 3 Division of Geriatric Gastroenterology, The First Affiliated Hospital of Kunming Medical University, Kunming, Yunnan, China; 4 Yunnan Key Laboratory of Primate Biomedical Research, Kunming, Yunnan, China

**Keywords:** aging, gut microbiota, intestinal inflammation, metabolomics, rhesus macaques

## Abstract

**Introduction:**

The gut microbiota and its metabolites change dynamically with age, but their trajectories and links to healthy aging are unclear, hindering the development of related interventions.

**Methods:**

We utilized captive rhesus macaques across three age groups, conducting 16S sequencing and targeted metabolomics analysis of fecal matter.

**Results:**

*Lactobacillus* predominated in young monkeys. Old adult monkeys exhibited the most severe intestinal inflammation, matching the dominance of Proteobacteria, *Prevotella*, and *Fusobacterium*. In oldest monkeys, the gut microbiota tended to be dominated by the short-chain fatty acid-producing *Butyricicoccus* and *Christensenellaceae*. Twenty-one metabolites gradually decrease with age, including omega-3 fatty acids, aspartic acid, ornithine, and TCA cycle-related metabolites. The urea cycle, glycine/serine metabolism, and arginine/proline metabolism undergo age-related changes, likely reflecting altered intestinal microbial metabolic activity and potentially affecting hepatic ammonia metabolism.

**Discussion:**

Ornithine or a-ketoglutarate supplementation mitigates cellular senescence in liver cells and bone marrow mesenchymal stem cells. Our findings elucidate the dynamic changes of gut microbiota and metabolites with age, providing critical support for the use of rhesus monkeys as an animal model to study the role of gut microbiota in human aging.

## 1 Introduction

Aging is characterized by progressive decline in physiological functions, including metabolic dysfunction, immune senescence, and chronic inflammation, ultimately leading to organ deterioration and increased susceptibility to aging-related diseases (Adav and Wang, 2021). Understanding the biological mechanisms of aging is essential for developing strategies to extend healthspan (O’Toole and Jeffery, 2015). The human gut harbors more than 100 trillion microorganisms, collectively regarded as a microbial organ that exerts profound influences on host physiology (Ling et al., 2022). Accumulating evidence indicate that alterations in the assembly, structure, and dynamics of human gut microbiota characterize age-related microbial dysbiosis, which exhibits a profound cross-sectional association with the remodeling of host metabolic and immune system functions during aging (Gill et al., 2006).

The gut microbiome is at the core of many age-related changes, and age-related processes may influence the gut microbiota and its associated metabolic alterations (Mohajeri et al., 2018). Early in life, delivery mode, maternal microbiota, and infant feeding shape a relatively simple ecosystem dominated by Bifidobacteria (Claesson et al., 2011; Mohajeri et al., 2018). Gut microbiome abundance and diversity increase from birth until around 12 years of age, stabilize relatively through adulthood, and then decline with aging (Lynch and Pedersen, 2016). In elderly individuals (> 70 years), gut microbial structure typically shifts toward increased *Bacteroidetes* and reduced *Firmicutes* compared to young adults. Longevity phenotypes show further divergence: centenarians display distinct rearrangements, including relative enrichment of *Bifidobacterium* despite its general decline in the elderly, suggesting a potential role in healthy aging (Depp and Jeste, 2006). Other genera also exhibit age-stratified patterns, *Streptococcus* is more prevalent in elderly populations, but in typical elderly cohorts yet remains low in individuals with extreme longevity (> 105 years) (Arboleya et al., 2016). *Lactobacillus* increases with age in UK and Russian populations (Kashtanova et al., 2020; Odamaki et al., 2016; Verdi et al., 2018), but is decreased in Chinese elderly with osteoporosis (Li et al., 2019).

Metabolic dysfunction is a hallmark of aging. Age-associated accumulation of visceral fat is strongly associated with the exacerbation of persistent systemic inflammation, partly due to impaired flexibility in switching between fatty acid and glucose metabolism (Bhatti et al., 2016; Haslam and James, 2005). Frailty in elderly individuals is frequently linked to diminished energy synthesis resulting from tricarboxylic acid (TCA) cycle dysfunction and abnormal fatty acid metabolism (Gonzalez-Freire et al., 2020). Furthermore, disruptions in amino acid metabolism accompany liver function decline, and liver aging has been identified as a key contributor to the deterioration of multiple organs in human (Kiourtis et al., 2024). Intestinal microbiota is closely associated with host metabolism, including energy balance, glucose homeostasis, and lipid metabolism (Sonnenburg and Bäckhed, 2016), while microbial dysbiosis is increasingly recognized as a key correlate of metabolic disorders. Fecal microbiota transplantation studies demonstrate that gut microbes can transfer metabolic phenotypes across hosts (Koutnikova et al., 2019; Vrieze et al., 2012), underscoring the potential of gut microbiota- and metabolite- targeted interventions to restore metabolic health and delay aging.

Despite substantial progress, research on human gut microbiota across the lifespan is limited by inter-individual heterogeneity related to genetics, environment, lifestyle, physiological conditions, and medical history (Zapata and Quagliarello, 2015). Moreover, there is no consensus on how to define healthy versus unhealthy aging, though long-lived individuals represent the most successful examples of healthy aging. With advances in biotechnology, particularly stem cell–based approaches, interventions to promote healthy aging are rapidly expanding. When evaluating such interventions in animal models, the influence of gut microbiota on host metabolism and immunity must be carefully considered (Cătoi et al., 2020). Suitable animal models are therefore essential to disentangle the complex interactions among microbiota, metabolism, and aging. The rhesus macaque (Macaca mulatta) represents one of the most translationally relevant models owing to its genetic, physiological, and immunological similarity to humans. Importantly, captive non-human primates harbor “humanized” gut microbiota that closely resembles that of humans, making them particularly valuable for aging research (Clayton et al., 2016; Yan et al., 2022).

In this study, we investigated gut microbiota and fecal metabolites in captive rhesus monkeys across three age groups: young, old adult and oldest. By integrating 16S rRNA sequencing with targeted metabolomics, we aimed to elucidate age-related microbial and metabolic dynamics, uncover potential microbiota–metabolite interactions, and provide insights into strategies for promoting healthy aging and longevity.

## 2 Materials and methods

### 2.1 Animal

A total of 24 rhesus monkeys were included in this study, comprising six young (7 years), nine old adults (12–13 years), and nine oldest (21–25 years) individuals (Supplementary Table 1). All animals were individually caged under controlled environmental conditions (12 h light/12 h darkness cycle; temperature 18–26 °C; humidity 40%–70%). Monkeys were fed twice daily and received fresh fruits and vegetables (apples, bananas, pears, onions, cabbage) once daily as dietary supplementation. All animal procedures were approved by the Institutional Animal Care and Use Committee of Kunming University of Science and Technology (LPBR-2020-07), and were performed in accordance with the Guide for the Care and Use of Laboratory Animals (8th edition).

### 2.2 Sample collection, tissue processing, and DNA extraction

Fresh fecal samples were collected from the 24 rhesus monkeys in sterile tubes, immediately transported to the laboratory on ice, and stored at −80 °C until use. Microbial genomic DNA was extracted from each fecal sample using the MoBioPowerSoil^®^ DNA Extraction Kit (Arlsbad, CA, USA) following the manufacturer’s protocol. DNA concentration was quantified using the Qubit^®^ DNA Assay Kit in Qubit^®^ 2.0 Flurometer (Life Technologies, CA, USA).

For the purpose of histopathological evaluation, fresh colonic tissue specimens (specifically targeting the proximal ascending colon) were opportunistically harvested immediately following the unexpected or accidental death of macaques across the three age cohorts. To ensure scientific validity, any animals with a history or gross necropsy evidence of primary gastrointestinal disease were strictly excluded from this histological subset. To arrest autolysis and preserve fine mucosal architecture, the tissue samples were gently irrigated with sterile phosphate-buffered saline (PBS) to clear luminal contents, followed by immediate immersion in 4% paraformaldehyde (PFA) for an optimized fixation period of 24–48 h. Post-fixation, the tissues were subjected to dehydration via a graded ethanol series, cleared in xylene, and subsequently embedded in paraffin wax. Using a rotary microtome, the paraffin blocks were sectioned at a standardized thickness of 5 μm. The resulting sections were mounted on glass slides, deparaffinized, rehydrated, and stained utilizing standard Hematoxylin and Eosin (H&E) protocols.

### 2.3 Amplicon generation

The 16S rRNA genes of distinct regions (16S V3-V4) were amplified using specific primers (16S V3-V4, 341F-806R; 16S V3, 528F-706R) with a barcode. All PCRs were carried out with 15 mL of Phusion high-fidelity PCR master mix (New England Biolabs, USA), 0.2 mM forward and reverse primers, and about 10 ng template DNA. Thermal cycling consisted of initial denaturation at 98 °C for 1 min, followed by 30 cycles of denaturation at 98 °C for 10 s, annealing at 50 °C for 30 s, and elongation at 72 °C for 30 s.

### 2.4 PCR product quantification and qualification

The same volume of 1× loading buffer (containing SYBR green) was mixed with PCR products, and electrophoresis on a 2% agarose gel was carried out for detection. PCR products were mixed in equidensity ratios. Then, mixtures of PCR products were purified with the Qiagen gel extraction kit (Qiagen, Germany).

### 2.5 Library preparation and sequencing

Sequencing libraries were generated using the TruSeq DNA PCR-free sample preparation kit (Illumina, USA) following the manufacturer’s instructions, and index codes were added. Library quality was assessed with a Qubit 2.0 fluorometer (Thermo Scientific, USA) and Agilent Bioanalyzer 2100 system (Agilent Technologies, USA). Libraries were then sequenced on an Illumina NovaSeq platform, and 250-bp paired-end reads were generated.

### 2.6 Analysis of fecal metabolites

Frozen fecal samples were thawed in an ice bath to minimize degradation. Approximately 50 mg of each sample were homogenized with 300 μL of NaOH (1 M) using a homogenizer (BB24, Next Advance, Inc., Averill Park, NY, USA). To monitor extraction efficiency and analytical variations, stable isotope-labeled internal standards were spiked into the samples prior to homogenization. The homogenates were then centrifuged at 4,000 × *g* for 20 min at 4 °C (Microfuge 20R, Beckman Coulter, Inc., Indianapolis, IN, USA). From each sample, 200 μL of supernatant was transferred into an autosampler vial (Agilent Technologies, Foster City, CA, USA). The residue was further extracted with 200 μL of cold methanol, homogenized, and centrifuged again; 167 μL of this secondary supernatant was combined with the first extract in the same vial.

Derivatization was carried out using a robotic multi-purpose sampler MPS2 with dual heads (Gerstel, Muehlheim, Germany). Briefly, 20 μL of methyl chloroformate (MCF) was added to each extract, followed by vigorous vortexing for 30 s. A second aliquot of 20 μL MCF was then added for repeat derivatization. Phase separation was achieved by sequential addition of 400 μL of sodium bicarbonate solution (50 mM). Samples were centrifuged at 4,000 × *g* for 20 min at 4 °C, and the bottom chloroform layer was carefully transferred by the robotic preparation station to an autosampler vial preloaded with approximately 25 mg of anhydrous sodium sulfate. After shaking at 450 × *g* for 20 min at 4 °C, the treated extract was transferred to a fresh autosampler vial for injection.

Metabolite profiling was performed using a gas chromatography coupled to a time-of-flight mass spectrometry (GC-TOFMS) system (Pegasus HT, Leco Corp., St. Joseph, MO, USA) operating in electron ionization (EI) mode. Instrument optimization was performed every 24 h. Raw data were processed using a proprietary software XploreMET (v2.0, Metabo-Profile, Shanghai, China) for automatic baseline denoising, smoothing, peak picking, and peak signal alignment. To ensure the robustness of the metabolic profiling and correct for potential batch effects, pooled quality control (QC) samples were prepared by mixing equal aliquots from all biological samples and were injected at regular intervals throughout the analytical sequence for QC-based non-linear data normalization. Metabolite identification was performed by matching accurate mass and retention indices against the Human Metabolome Database (HMDB) and the proprietary XploreMET database, achieving high-confidence annotations in strict adherence to the Metabolomics Standards Initiative (MSI) Level 1 or Level 2 guidelines. The analytical consistency was further validated by the tight clustering of QC samples in the PCA plot and high correlation coefficients (*r* > 0.985) among QC injections (Supplementary Figure 2).

### 2.7 16 S rRNA sequencing analysis

A total of 24 fecal samples from rhesus monkeys were subjected to 16S rRNA gene sequencing. Raw sequencing data were processed using the Mothur pipeline following the standard operating procedure. Briefly, sequences were quality-filtered, aligned, and clustered into operational taxonomic units (OTUs) at a 97% sequence identity threshold. Taxonomic classification was performed against the SILVA database using a confidence threshold ranging from 0.8 to 1.0. Microbial community composition was summarized at multiple taxonomic levels, including kingdom, phylum, class, order, family, genus, and species. To minimize biases introduced by uneven sequencing depth, all samples were rarefied to the minimum sequencing depth observed across samples. Subsequent diversity analyses were conducted based on the rarefied OTU table. Alpha diversity was assessed using the Shannon index and Simpson index to evaluate community richness and evenness. Beta diversity was analyzed using principal coordinates analysis (PCoA) and non-metric multidimensional scaling (NMDS) based on distance matrices calculated in the vegan and ape R packages. Indicator species analysis was conducted using the labdsv R package to identify taxa significantly associated with specific time points. Functional profiles of indicator taxa were predicted using Tax4Fun, enabling inference of potential microbial metabolic functions.

### 2.8 Integrated microbiome-metabolome analysis

To explore the associations between microbial taxa and metabolites, Pearson correlation coefficients were calculated between indicator microorganisms and differential metabolites. Significant correlations were used to construct microbiome–metabolome interaction networks, which were visualized to reveal potential functional relationships between microbial communities and host metabolic profiles.

### 2.9 *In vitro* experiment of human liver cells

Cell culture Human liver cells (L-02, EouCell, China) were cultured in RFMI 1640 Medium (Gibco) supplemented with 20% heat-inactivated fetal bovine serum (Sigma) and 1% penicillin/streptomycin (HyClone). Liver cells were cultured at 37 °C in a humidified incubator with 5% CO_2_ and passaged at 90% confluence.

Senescence-associated β-galactosidase (SA β-gal) staining L-02 cells were treated H_2_O_2_ (400 μmol/L) for 24 h, followed by ornithine supplementation (10 mM, Sigma-Aldrich) for 48 h. Then, SA-β-Gal staining was performed to assess the aging and proliferation ability of liver cells. SA-β-gal staining was performed using a commercial Kit (Beyotime Biotech Inc, China). Briefly, cells were washed with PBS, fixed for 15 min, and incubated with staining solution in a CO_2_-free incubator at 37 °C. The staining reaction was terminated at 10 h when blue–green staining appeared. Images were captured at three random fields per slide using a Leica DMIL LED microscope (Leica, Wetzlar, Germany), and the percentage of SA-β-gal-positive cells was calculated relative to the total number of cells, with all experiments conducted using three independent biological replicates (*n* = 3).

EdU assay: Cell proliferation was assessed using the EdU Cell Proliferation Kit with Alexa Fluor 488 (Beyotime Biotech Inc, China). After incubation with EdU working solution for 8 h, cells were fixed with 4% paraformaldehyde for 15 min, permeabilized with 0.5% Triton X-100 for 15 min, and subjected to the Click reaction for 30 min in dark. Cell nuclei were counterstained with Hoechst 33342 for 10 min in dark. After being washed three times with PBS, images were captured under a fluorescence microscope (Leica, Wetzlar, Germany), and EdU-positive cells was quantified using ImageJ (National Institutes of Health, USA), with all experiments conducted using three independent biological replicates (*n* = 3).

Quantitative RT-PCR: Total RNA was extracted from cultured liver cells using Trizol (Invitrogen), and purity/concentration was determined with a NanoDrop 2000 (Thermo Fisher Scientific). One microgram cDNA of each sample was obtained using the PrimeScript RT reagent Kit with gDNA Eraser (Takara). The real-time PCR was performed using SYBR Premix Ex Taq II (Takara) in CFX96 Real-Time System (Bio-Rad), and the relative expression of target gene was normalized by housekeeping gene (Gapdh) using a 2^–ΔΔCt^ method subsequently. The primers used for qPCR are presented in Supplementary Table 1.

### 2.10 *In vitro* experiment of bone marrow mesenchymal stem cells from macaques

Cell culture Following intramuscular anesthetization with a combination of ketamine (15 mg/Kg) and xylazine (0.5 mg/Kg), primary bone marrow mesenchymal stem cells (BMSCs) were obtained from aged macaques via iliac crest bone marrow aspiration. During the anesthetic procedure, vital physiological parameters including heart rate, respiratory rate, blood oxygen saturation, and body temperature were continuously monitored to ensure the safety and appropriate anesthetic depth. Cells were cultured in Dulbecco’s Modified Eagle Medium (DMEM; Gibco) supplemented with 10% heat-inactivated fetal bovine serum (sigma) and 1% penicillin/streptomycin (HyClone). BMSCs were passaged at 90% confluence.

Assessment of proliferation and aging: BMSCs were treated with α-ketoglutarate (α-KG, 4 mM; Sigma) for 48 h. Senescence and proliferation were evaluated by SA-β-Gal and EdU staining, respectively, following the protocols described above.

Bulk RNA sequencing: Total RNAs was extracted from aged BMSCs with or without αKG treatment using Trizol (Invitrogen). Sequencing libraries were generated using NEBNext^®^ UltraTM RNA Library Prep Kit (NEB, USA) on an Illumina NovaSeq platform (150 bp paired-end). Raw reads were processed with applied fastp (version 0.23.2) for quality control, and gene expression was quantified using feature-Counts (version 2.0.3). Data were then visualized and analyzed using custom R scripts in combination with the Bioconductor package DESeq2 (version 1.44.0). Batch effects were corrected prior to downstream analyses, and heatmaps were generated with pheatmap (version 1.0.12) to illustrate gene expression pathways. Upregulated genes were defined as those with padj < 0.05 and log2FoldChange > 0.5, while downregulated genes were defined as those with padj < 0.05 and log2FoldChange < −0.5.

### 2.11 Statistical analysis

Experiments were not randomized. All statistical analyses were conducted using R software, GraphPad Prism, and IBM SPSS Statistics 26. Prior to statistical comparisons, data distribution and normality were rigorously evaluated using the Shapiro–Wilk test, and homogeneity of variances was assessed using Levene’s test. For normally distributed data with equal variances (e.g., differential metabolites and *in vitro* cell assays), statistical significance among the three age groups was determined using a one-way analysis of variance (ANOVA) followed by Tukey’s honestly significant difference (HSD) *post-hoc* test for multiple pairwise comparisons. For non-normally distributed data (e.g., microbiome alpha diversity indices), the non-parametric Kruskal–Wallis test was applied.

To control for false positives across high-dimensional omics datasets, multiple testing correction was performed using the Benjamini–Hochberg false discovery rate (FDR) method, with statistical significance strictly defined as an FDR-adjusted *q*-value < 0.05. Furthermore, effect sizes are systematically reported to demonstrate the magnitude of differences: log2 fold change (log2FC) was calculated for differential metabolites, and Enrichment Ratios were calculated for metabolic pathways. Pearson correlation coefficients were used to measure associations between indicator microbial taxa (identified via the labdsv R package) and specific metabolites. Errors and error bars represent the standard deviation (SD) from independent samples of each group, and the exact sample sizes (*n*) are explicitly denoted in all respective figure legends.

## 3 Results

### 3.1 Age-associated alterations in gut microbial diversity and composition in rhesus monkeys

**FIGURE 1:**
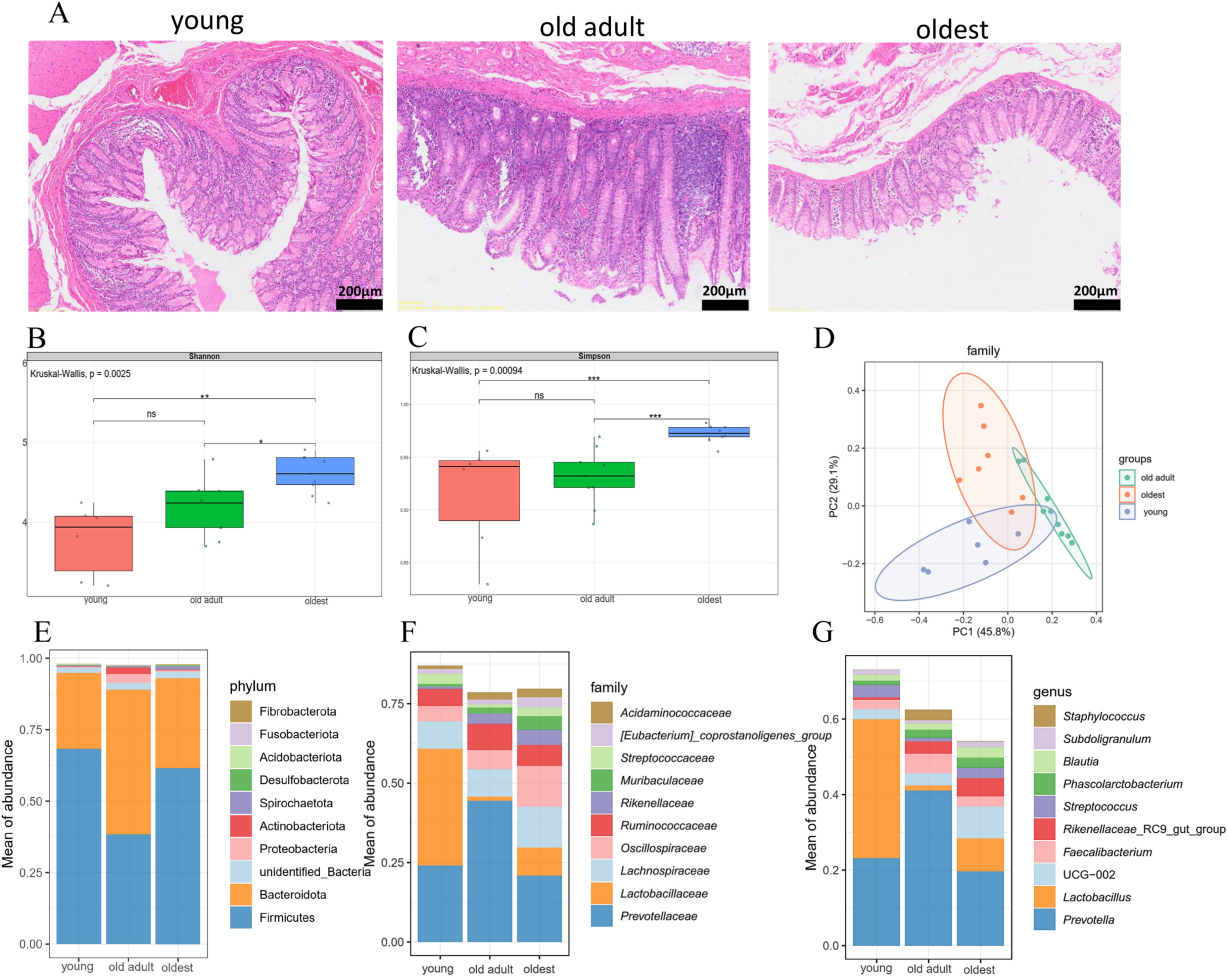
Age-related changes in colonic histology, microbial diversity, and taxonomic composition of rhesus monkeys. **(A)** Pathological analysis of colon in three age groups of macaques. **(B,C)** Alpha diversity analysis including Shannon index and Simpson index. Statistical significance among the three groups was evaluated using the non-parametric Kruskal–Wallis test followed by Benjamini–Hochberg false discovery rate (FDR) correction. *FDR *q* < 0.05; **FDR *q* < 0.01; ***FDR *q* < 0.001; ns, not significant. **(D)** Difference in beta diversity between three age groups by PCoA analysis. The composition of gut microbiota analysis at the phylum level **(E)**, family level **(F)**, and genus level **(G)** Exact sample sizes are *n* = 6 (young), *n* = 9 (old adult), and *n* = 9 (oldest). For visual clarity, rare taxa with extremely low relative abundances are not displayed; therefore, the sum of the relative abundances shown does not equal 100%.

Histological examination of colon tissues revealed distinct age-related morphological changes. Compared to young monkeys, old adult monkeys displayed significant inflammatory cell infiltration, whereas the oldest group exhibited loosely arranged goblet cells in the intestinal wall but no apparent inflammatory infiltration (Figure 1A). Analysis of microbial diversity indicated significant aging-associated shifts. Analysis of microbial diversity indicated significant aging-associated shifts. Based on the Kruskal–Wallis test with Benjamini–Hochberg false discovery rate (FDR) correction, the oldest monkeys displayed significantly higher alpha diversity than both the young monkeys (Shannon: FDR *q* = 0.002; Simpson: FDR *q* = 0.001) and the old adult monkeys (Shannon: FDR *q* = 0.016; Simpson: FDR *q* = 0.001). However, no significant differences in alpha diversity were observed between the young and old adult groups (Shannon: FDR *q* = 0.181; Simpson: FDR *q* = 0.864) (Figures 1B,C). Furthermore, principal coordinate analysis (PCoA) based on Bray–Curtis distances showed clear separation of gut microbial community structure across the three age groups (PERMANOVA, adjusted *R*^2^ = 0.218, *P* = 0.001; Figure 1D).

Taxonomic profiling demonstrated marked compositional differences. At the phylum level, the abundance of *Firmicutes* was significantly decreased in old adult monkeys compared to young and oldest monkeys (vs. young: *p* = 0.0008, FDR = 0.0048; vs. oldest: *p* = 0.0001, FDR = 0.0007), whereas the abundance of Bacteroidota and Proteobacteria were increased (*p* = 0.0016, FDR = 0.0051 and *p* = 0.0008, FDR = 0.0048 vs. young, respectively). In the oldest monkeys, the abundance of *Firmicutes* also declined relative to young monkeys, while Spirochaetota displayed the highest relative abundance (Figure 1E). At the family level, the relative abundance of Lactobacillaceae was significantly decreased in both old adult and oldest monkeys (vs. old adult: *p* = 0.0004, FDR = 0.0080; vs. oldest: *p* = 0.0008, FDR = 0.0304), whereas Oscillospiraceae, Rikenellaceae and Muribaculaceae were significantly increased the oldest monkeys compare to both young and old adult monkeys (vs. young: *p* = 0.0016–0.0076, FDR = 0.0304–0.0585; vs. old adult: *p* = 0.0008–0.0078, FDR = 0.0023–0.0178) (Figure 1F). At the genus level, the abundance of *Prevotella* was markedly increased in the old adult monkeys (vs. young: *p* = 0.0004–0.0008, FDR = 0.0094), while *Lactobacillus* was significantly decreased in both old adult and oldest monkeys compared to young monkeys (vs. old adult: *p* = 0.0004, FDR = 0.0094; vs. oldest: *p* = 0.0008, FDR = 0.0357) (Figure 1G).

### 3.2 Indicator taxa across different age stages

**FIGURE 2:**
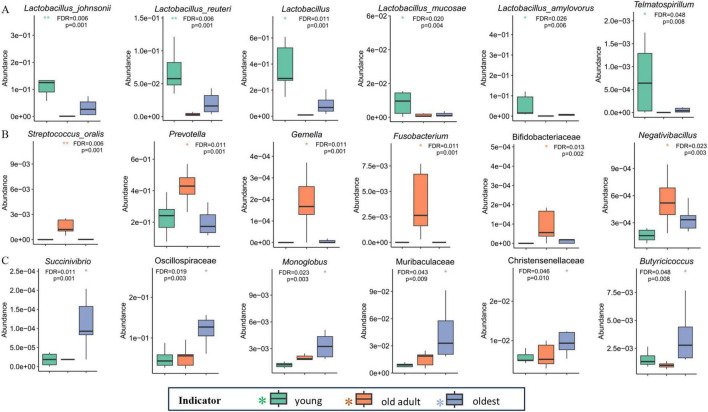
Age-associated genus- and family-level indicator microbiota in rhesus monkeys. **(A)** Indicator taxa of young monkeys. **(B)** Indicator taxa enriched in old adult monkeys. **(C)** Indicator taxa enriched in oldest monkeys. Indicator gut microbial taxa were identified using the IndVal function. Statistical significance was evaluated with Benjamini–Hochberg FDR correction. *FDR *q* < 0.05; **FDR *q* < 0.01. Exact sample sizes are *n* = 6 (young), *n* = 9 (old adult), and *n* = 9 (oldest). The displayed taxa represent the entire set of significant indicator species identified under the strict criterion of FDR *q* < 0.05.

Indicator species can reveal alterations in an ecosystem’s biological state through its presence, absence, or population density (Chiarucci and Piovesan, 2020). Distinct age-associated indicator taxa were identified in the macaque gut microbiota. In the young group, *Lactobacillus* and its species (*L. amylovorus*, *L. johnsonii*, *L. mucosae*, *L. reuteri*), together with *Telmatospirillum*, were enriched (Figure 2A). In the old adult group, *Prevotella*, Negativibacillus, *Gemella*, *Fusobacterium*, Bifidobacteriaceae, and *Streptococcus oralis* showed significantly increases (Figure 2B). In the oldest group, Succinivibrio, *Butyricicoccus*, *Christensenellaceae*, Muribaculaceae, Monoglobus, and Oscillospiraceae were significantly elevated (Figure 2C). Functional enrichment analysis revealed that the gut microbiota in old adult monkeys was primarily involved in the degradation of aromatic compounds and the biosynthesis of ansamycins, whereas, in the oldest monkeys it was enriched in pathways related to arginine synthesis and oxidative phosphorylation (Supplementary Figure 1A).

### 3.3 Age dependent remodeling of metabolic patterns in rhesus monkeys

**FIGURE 3:**
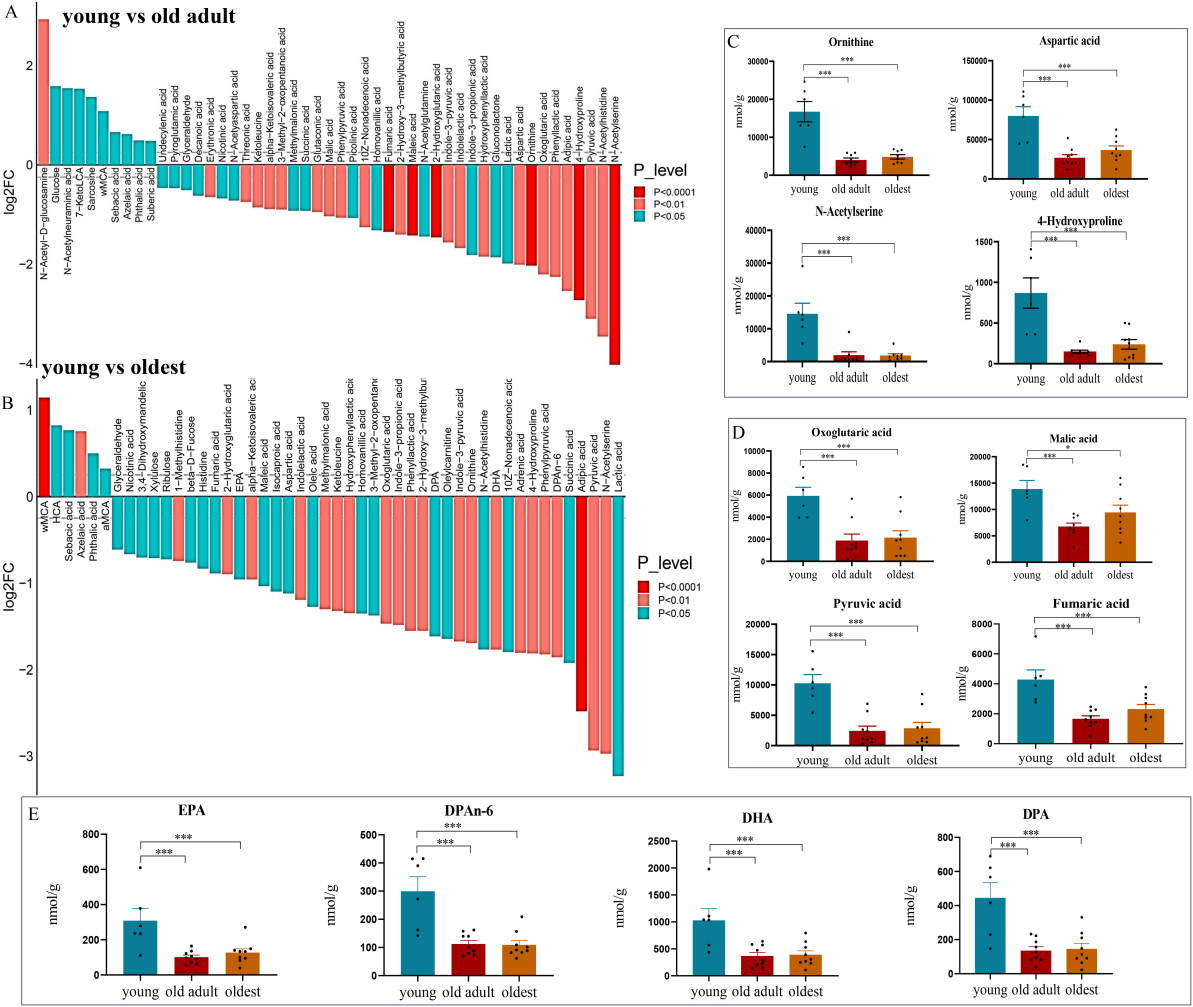
Differential metabolic profiles of monkey feces across age stages. **(A)** log2FC is a positive number indicating metabolites are significantly increased in old adult macaques, log2FC is a negative number indicating metabolites are significantly elevated in young macaques. **(B)** log2FC is a positive number indicating metabolites are significantly increased in oldest macaques, log2FC is a negative number indicating metabolites are significantly elevated in young macaques. **(C)** Concentration of four amino acids (Ornithine, Aspartic acid, N-Acetylserine, 4-Hydroxyproline) in fecal samples from three groups of monkeys. **(D)** Concentration of four organic acid (Oxoglutaric acid, Malic acid, Pyruvic acid, Fumaric acid) in fecal samples from three groups of monkeys. **(E)** Concentration of four fatty acid (EPA, DPAn-6, DHA, DPA) in fecal samples from three groups of monkeys. Data are presented as mean ± SD. Statistical significance among the three groups was determined using one-way ANOVA followed by Tukey’s HSD *post-hoc* test. *p < 0.05; **p < 0.01; ***p < 0.001. Exact sample sizes are *n* = 6 (young), *n* = 9 (old adult), and *n* = 9 (oldest), as indicated by the individual scatter dots.

Ultra-performance liquid chromatography coupled to tandem mass spectrometry (UPLC-MS/MS) identified pronounced age-dependent shifts in fecal metabolite profiles. In total, 49 metabolites distinguished young from old adult monkeys (Figure 3A), 48 metabolites distinguished young from oldest monkeys (Figure 3B), and 4 metabolites distinguished old adult from oldest monkeys (Supplementary Figure 1B). Metabolites associated with the tricarboxylic acid (TCA) cycle (Oxoglutaric acid, Malic acid, Pyruvic acid, Fumaric acid), amino acids (Ornithine, Aspartic acid, N-Acetylserine, 4-Hydroxyproline) and omega-3 fatty acids (EPA, DHA, DPA, DPAn-6) were significantly reduced in both the old adult and oldest monkeys (One-way ANOVA, Tukey’s HSD *post-hoc* test, all *p* < 0.05). However, compared to the old adult monkeys, the concentrations of these metabolites did not continue to decline in the oldest monkeys, instead, they showed a gradual increase (Figures 3C–E). This pattern is similar to the alterations observed in the gut microbiota of oldest monkeys, which is a trend of metabolic response recovery toward youthfulness.

### 3.4 Age-associated dynamics of metabolites and corresponding metabolic pathways

**FIGURE 4:**
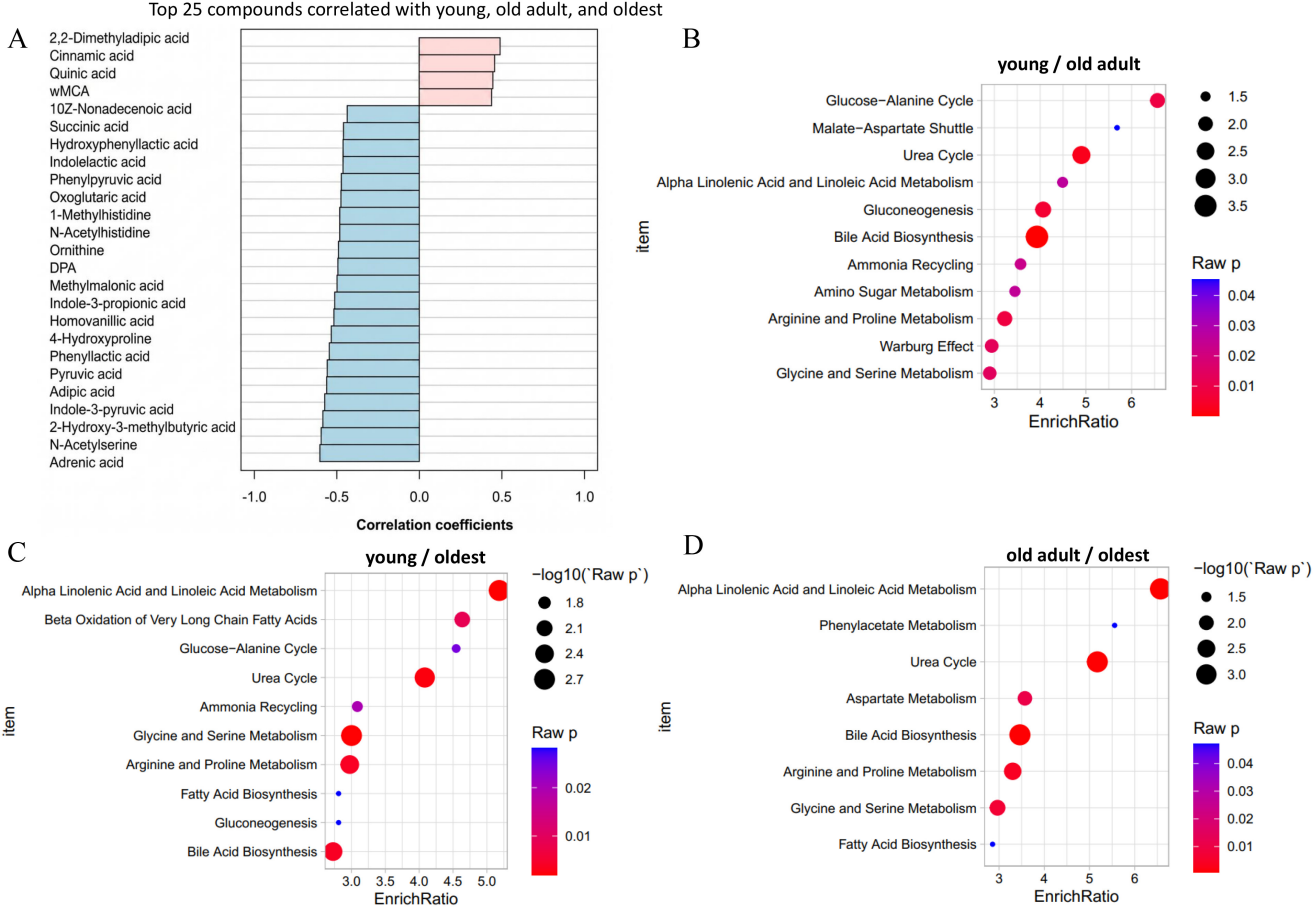
Age-related metabolite dynamics and associated pathway analysis. **(A)** Temporal changes of metabolites across the three age groups. Twenty-one metabolites gradually decreased with age, while four metabolites increased. **(B)** Comparison of differential metabolic pathways between young and old adult macaques. **(C)** Comparison of differential metabolic pathways between young and oldest macaques. **(D)** Comparison of differential metabolic pathways between old adult and oldest macaques. Exact sample sizes analyzed were *n* = 6 (young), *n* = 9 (old adult), and *n* = 9 (oldest).

To investigate the dynamic relationship between gut microbiota and metabolites during aging, we conducted metabolic pathway enrichment analyses. Twenty-one metabolites gradually decreased with age, including oxoglutaric acid, DPA, 4-hydroxyproline and pyruvic acid, whereas four metabolites gradually increased, including 2,2-Dimethyladipic, cinnamic acid, quinic acid and wMCA (Figure 4A). Metabolic pathway analysis of differential metabolites revealed age-associated impairments in key metabolic processes, including energy synthesis (e.g., glucose-alanine cycle, malate-aspartate shuttle), amine metabolism (urea cycle, ammonia recycling), bile acid synthesis, and alpha-linolenic acid and linoleic acid metabolism. Additionally, abnormal amino acid metabolism, particularly affecting glycine-serine and arginine-proline pathways, was also observed (Figures 4B–D). Collectively, these findings suggest that aging is associated with progressive mitochondrial dysfunction and disruption of critical metabolic pathways.

### 3.5 Age-related associations between gut microbiota and fecal metabolites

**FIGURE 5:**
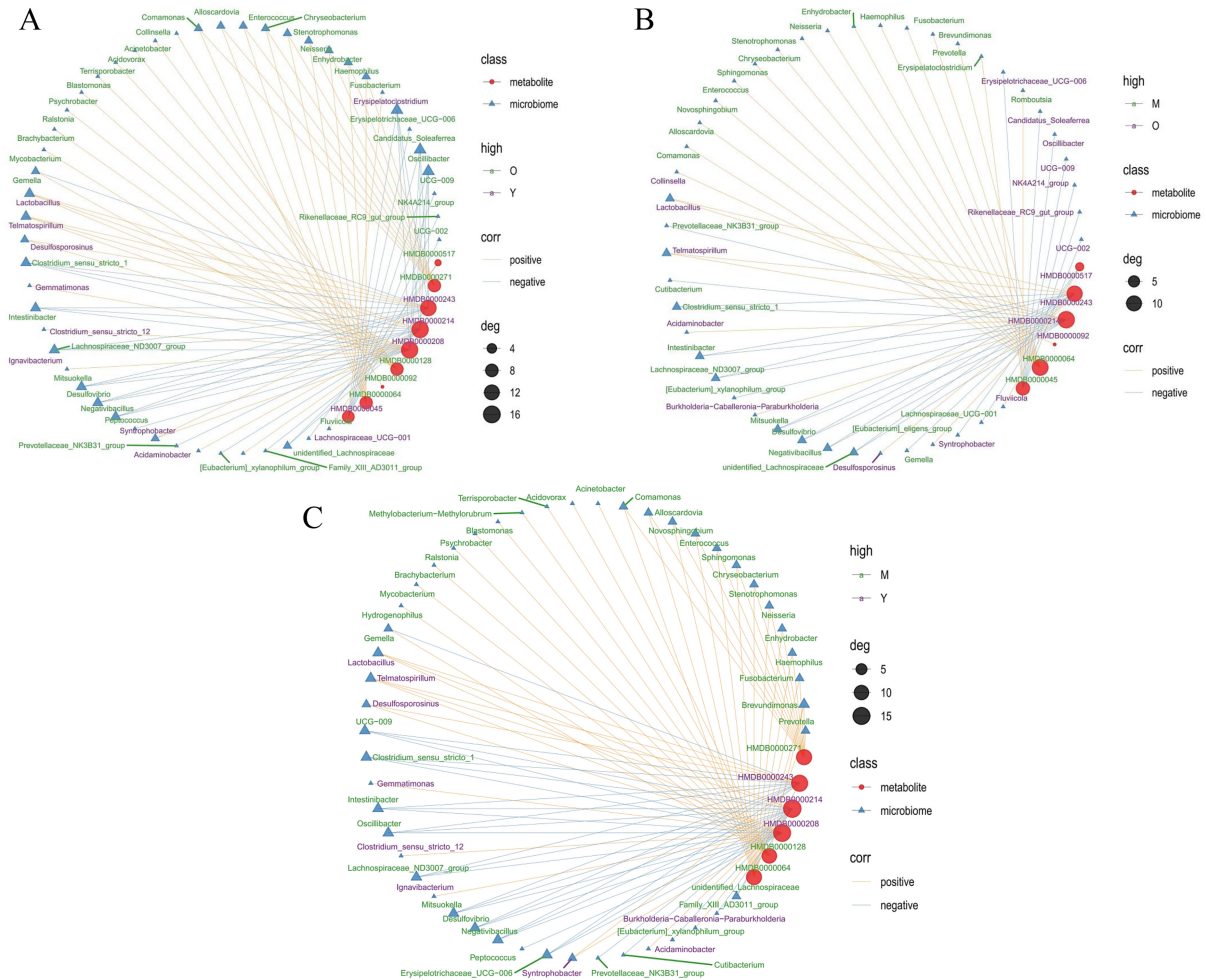
Associations between microbial indicator taxa and specific metabolic pathway intermediates. **(A)** Correlation analysis between differential metabolites and indicator microbiota in young versus old adult macaques. **(B)** Correlation analysis between differential metabolites and indicator microbiota in young versus oldest macaques. **(C)** Correlation analysis between differential metabolites and indicator microbiota in old adult versus oldest macaques. Associations were evaluated using Pearson correlation coefficients. Exact sample sizes are *n* = 6 (young), *n* = 9 (old adult), and *n* = 9 (oldest).

Integrated analysis of differential metabolites and indicator microbiota demonstrated that *Lactobacillus* and *Telmatospirillum* were positively correlated withTCA cycle via pyruvic acid (HMDB0000243) and oxoglutaric acid (HMDB0000208), thereby enhancing ATP production. *Lactobacillus* also showed a strong positive association with ornithine synthesis (HMDB0000214), facilitating its role in amine metabolism (Figures 5A,B). Meanwhile, *Prevotella* and *Fusobacterium* were found to be positively associated with intermediates of glycine metabolism, Including Sarcosine (HMDB0000271) and creatine (HMDB0000064) (Figures 5B,C).

### 3.6 Ornithine and α-ketoglutarate ameliorate liver cell and BMSC aging *in vitro*

**FIGURE 6:**
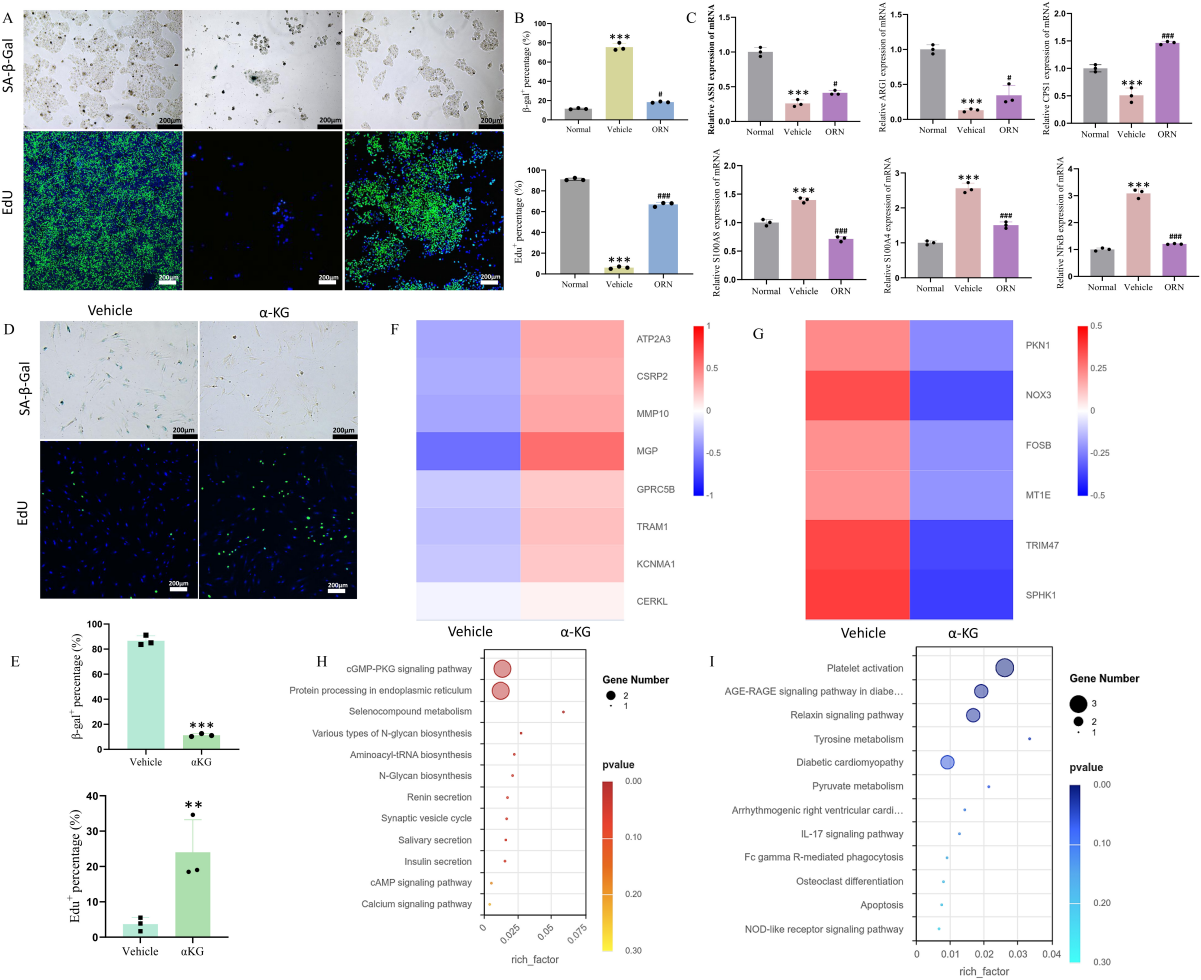
Effects of ornithine and α-ketoglutarate on liver cell and BMSC aging. **(A)** SA-β-Gal and EdU staining of human liver cells. Scale bar: 200 μm. Blue fluorescence corresponds to the Hoechst 33342 nuclear stain, and green fluorescence represents the Alexa Fluor 488-labeled EdU. **(B)** Quantification of SA-β-Gal-positive and EdU-positive liver cells (*n* = 3). **(C)** Quantitative RT-PCR analysis of urea cycle and aging-related genes in liver cells (*n* = 3). **(D)** SA-β-Gal and EdU staining of BMSCs. Blue fluorescence corresponds to the Hoechst 33342 nuclear stain, and green fluorescence represents the Alexa Fluor 488-labeled EdU. Scale bar: 200 μm. **(E)** Quantification of SA-β-Gal-positive and EdU-positive BMSCs (*n* = 3). **(F)** Heatmap showing genes significantly upregulated in α-KG-treated BMSCs. **(G)** Heatmap showing genes significantly downregulated in α-KG-treated BMSCs. **(H)** KEGG pathways significantly upregulated after α-KG treatment. **(I)** KEGG pathways significantly downregulated after α-KG treatment. Data in bar plots are presented as mean ± SD of independent biological replicates. Statistical significance was determined using one-way ANOVA followed by Tukey’s HSD *post-hoc* test. *p < 0.05; **p < 0.01; ***p < 0.001 compared to the normal group. #*p* < 0.05; ##*p* < 0.01; ###*p* < 0.001 compared to the vehicle group. Exact sample sizes (*n*) are indicated by individual scatter dots in the respective panels (*n* = 3).

Old adult monkey exhibited significant downregulation of metabolic pathways associated with liver aging, accompanied by a notable decrease in ornithine concentration. Because the liver serves as the primary metabolic interface of the gut-liver axis and ornithine is a critical substrate of the hepatic urea cycle, we selected human L-02 liver cells as a translationally relevant *in vitro* model. To evaluate whether ornithine could ameliorate cellular senescence, human normal liver cells were treated with hydrogen peroxide to induce senescence, followed by ornithine supplementation. Ornithine significantly reduced β-galactosidase levels while markedly increasing EdU expression in liver cells (Figures 6A,B). Furthermore, expression of urea cycle-regulated genes (ASS1, ARG1 and CPS1) increased, whereas aging and inflammation-related genes (S100A8, S100A4 and NFκB) were significantly downregulated (Figure 6C).

Given that TCA cycle dysfunction increases with age, whether intermediates in the TCA cycle could ameliorate aging was further investigated. We tested α-ketoglutarate (α-KG) on BMSCs from aged monkeys. α-KG treatment significantly reduced β-galactosidase levels in BMSCs and enhanced EdU expression (Figures 6D,E). Transcriptomic analysis of α-KG-treated BMSCs showed significant upregulation of osteogenesis-related (MGP, MMP10) and antioxidation genes (TRAM1, CERKL), whereas pro-inflammation and oxidative stress-related genes (PKN1, NOX3, TRIM47, SPHK1) were downregulated (Figures 6F,G). KEGG pathway enrichment analysis further demonstrated that α-KG suppressed IL-17 signaling and osteoclast differentiation while activating the cGMP-PKG signaling pathway (Figures 6H,I).

## 4 Discussion

In this study, we investigated dynamic changes in gut microbiota and fecal metabolites across three age groups of rhesus monkeys, aiming to elucidate their associations with aging and metabolic regulation. Histological examination revealed that old adult monkeys exhibited pronounced inflammatory infiltration in the colon, whereas the oldest monkeys primarily showed thinning of the intestinal wall and a reduction in goblet cell numbers without significant inflammation. Intestinal inflammation is a well-recognized contributor to unhealthy aging (DeJong et al., 2020). Consistent with findings in humans, where long-lived individuals exhibit higher gut microbial diversity than younger adults (Kong et al., 2016; Tuikhar et al., 2019), we observed that oldest monkeys also displayed the highest alpha diversity of gut microbiota. Moreover, both young and oldest monkeys harbored the highest levels of *Firmicutes* and the lowest levels of *Bacteroidetes*, a pattern paralleling that of human longevity cohorts, while a reduced *Firmicutes*/*Bacteroidetes* ratio is associated with metabolic disorders (Walters et al., 2014).

We further identified distinct gut microbial signatures across the three age groups in rhesus monkeys. Young monkeys were enriched in *Lactobacillus* species, including *L. johnsonii*, *L. amylovorus*, and *L. reuteri*. These species contribute to the short-chain fatty acid production and suppression of intestinal inflammation (Mager et al., 2020; Zheng et al., 2021). In contrast, the oldest monkeys exhibited microbial profiles closely resembling those of long-lived humans, characterized by enrichment of *Butyricicoccus* and *Christensenellaceae* (Biagi et al., 2016). *Butyricicoccus* has been shown to attenuate intestinal inflammation (Eeckhaut et al., 2013), while *Christensenellaceae* alleviates intestinal inflammation by regulating the transition of intestinal macrophages toward anti-inflammatory phenotype (Yao et al., 2024). Together, our results delineate distinct gut microbial trajectories across monkey aging, and the convergence of the microbiota of oldest monkeys with that of long-lived human underscores conserved microbial features, highlighting potential microbial targets for interventions to promote healthy aging.

Parallel to microbial changes, we identified fecal metabolite markers exhibiting age dynamic changes. Omega-3 polyunsaturated fatty acids (*n*-3 PUFAs), intermediate metabolites involved in the TCA cycle, and key amino acids such as ornithine, 4-hydroxyproline, and N-Acetylserine were significantly reduced in the old adult and oldest monkeys. However, these metabolites did not continue to decline in the oldest monkeys. In humans, epidemiological and randomized controlled trials have demonstrated a positive association between PUFAs and long-term health benefits, such as reduced risk of cardiovascular disease, decrease of morbidity, mortality, and protection against inflammatory conditions including arthritis and asthma (Aon et al., 2020; Baker et al., 2016). The intermediate metabolites (Oxoglutaric acid, Pyruvic acid) involved in the TCA cycle gradually decline with age, which may contribute to the diminished energy and physiological weakness observed in elderly individuals (Dent et al., 2019). The decline in ornithine is associated with liver aging, and supplementation can promote liver regeneration (Horvath et al., 2022), while 4-Hydroxyproline, a major component of collagen (Udenfriend, 1966), and is crucial for maintaining skeletal muscle function (Wohlgemuth et al., 2024). In summary, these findings indicate that age-related metabolites exhibit distinct associations with healthy aging, providing an observational foundation for exploring metabolic intervention strategies.

Four metabolic pathways were profoundly impacted by aging include the urea cycle, bile acid biosynthesis, glycine and serine metabolism, and arginine and proline metabolism. As the metabolic hub of the body (Timchenko, 2009), the liver communicates bidirectionally with the gut through the gut-liver axis. The gut microbiota participates in shaping the bile acid pool by converting primary bile acids into secondary bile acids, yet this diversity of the bile acid pool decreases with age (Collins et al., 2023). Impaired urea cycle function and abnormal ammonia recycling directly lead to hepatic ammonia toxicity (Lee et al., 2018). The production of ammonia is detoxified via the urea cycle with the participation of arginine and aspartate. Arginine was essential for efficient ammonia clearance and urea synthesis (Morris, 2002). Targeting glycine-serine metabolism in the liver can promote healthy aging and longevity, which is detoxified via the urea cycle with the participation of arginine and aspartate (Aon et al., 2020). Therefore, the depletion of urea cycle-related metabolites in feces primarily reflects alterations in the metabolic activity and dysbiosis of the gut microbiota. Given the close bidirectional communication of the gut-liver axis, these changes in the intestinal microenvironment might increase the ammonia detoxification burden on the liver. However, because our current study relies exclusively on fecal metabolomics, future investigations incorporating direct evaluations of plasma, bile, or liver tissue are required to definitively confirm whether hepatic ammonia clearance capacity is effectively impaired during macaque aging. *In vitro*, ornithine supplementation ameliorated L-02 liver cell senescence, accompanied by the transcriptional upregulation of urea cycle-related genes and downregulation of pro-inflammatory genes. Similarly, transcriptomic analysis revealed that α-KG supplementation in cultured BMSCs from aged monkeys improved cellular senescence, associated with the transcriptional downregulation of genes enriched in the IL-17 signaling pathway and apoptosis. Together, these observational findings highlight significant age-related microbiota-metabolite associations. While our *in vitro* data provide preliminary mechanistic insights, further *in vivo* interventional studies are needed to determine if targeting the gut microbiota and key metabolites represents a viable strategy to counteract age-related physiological decline and support healthy longevity.

Despite the valuable insights gained from this non-human primate model, several limitations of the current study must be acknowledged. First, the cross-sectional design restricts our ability to definitively distinguish true longitudinal aging trajectories from inherent cohort effects. Specifically, the attenuated inflammatory profiles and unique microbial signatures observed in the “oldest” macaque group may partially reflect survivor bias. Rather than demonstrating a universal “youth-like” reversal of aging, these extreme-aged individuals likely represent a highly resilient elite subgroup possessing intrinsic physiological and immunological advantages that allowed them to outlive their peers. Future longitudinal studies tracking the gut microbiota and metabolome of the same individuals over an extended timeframe are required to validate these temporal dynamics. Second, the study is constrained by a relatively small sample size and a lack of experimental randomization, which are common challenges driven by the extreme scarcity of geriatric macaque resources and ethical considerations. Moreover, while captive macaques harbor a “humanized” gut microbiota, their long-term exposure to standardized husbandry practices and specifically formulated diets exerts a profound selective pressure on microbial composition. This dietary and environmental convergence necessitates caution when extrapolating these microbial signatures to human populations, who experience vast dietary and lifestyle heterogeneity. Additionally, relying on a single-time-point fecal sampling limits our capacity to account for intra-individual microbial stability and potential circadian variations. Finally, there are notable limitations regarding our mechanistic validations. The metabolomics analysis was exclusively performed on fecal samples. Without concurrent plasma, bile, or hepatic metabolomic profiling, directly inferring systemic metabolic impairment or specific liver functions (such as hepatic ammonia clearance) from fecal data requires cautious interpretation. Furthermore, while the L-02 and BMSC lines serve as valuable tools for investigating hepatic metabolism and age-related osteoporosis, respectively, our *in vitro* 2D cell culture models cannot fully replicate the highly complex, three-dimensional physiological environment of the *in vivo* gut-liver axis. Additionally, the pharmacological doses (10 mM ornithine and 4 mM α-KG) required to effectively rescue cells in acute *in vitro* oxidative stress models exceed typical physiological baseline levels. Therefore, these *in vitro* results serve primarily as a preliminary mechanistic proof-of-concept rather than definitive physiological proof.

In conclusion, our integrative analysis of gut microbiota and metabolites across rhesus monkey aging reveals age-specific trajectories that parallel features of human longevity. We identified conserved microbial signatures, age-related changes in lipid and amino acid metabolism, and key gut–liver axis pathways shaping systemic aging. Experimental validation further highlights the potential of metabolites such as ornithine and α-ketoglutarate in alleviating cellular senescence. Our findings advance understanding of the microbiome–metabolism–aging nexus and provide a basis for microbiota- and metabolite-based strategies to promote healthy aging.

## Data Availability

The datasets presented in this study can be found in online repositories. The names of the repository/repositories and accession number(s) can be found below: https://ngdc.cncb.ac.cn/search/specific?db=bioproject&q=PRJCA045927, PRJCA045927.
