# Supplementary material for: Different ages of gut microbiota diversity and metabolic pattern in rhesus macaques
Source: Front Microbiol. 2026 Jul 28;17:1747272. doi: 10.3389/fmicb.2026.1747272 (PMC13460033; doi:10.3389/fmicb.2026.1747272)
Supplement: Supplementary file 1 [file Table_1.docx]

**Table S1. Animal information**

| **Animal** | **Group** | **Sex** | **Weight (Kg)** | **Age (years)** | **Health Status** | | **Diet** | **Housing Variables** |
| --- | --- | --- | --- | --- | --- | --- | --- | --- |
| 98340 | oldest | female | 15.2 | 23 | | Clinically healthy | Standard macaque chow + fruits/vegetables | 511-1  Individual cage, standard facility |
| 98090 | oldest | female | 14.8 | 23 | | Clinically healthy | Standard macaque chow + fruits/vegetables | 511-2  Individual cage, standard facility |
| 98380 | oldest | female | 16 | 23 | | Clinically healthy | Standard macaque chow + fruits/vegetables | 511-3  Individual cage, standard facility |
| 98018 | oldest | female | 15.9 | 23 | | Clinically healthy | Standard macaque chow + fruits/vegetables | 511-4  Individual cage, standard facility |
| 96061 | oldest | male | 12.3 | 25 | | Clinically healthy | Standard macaque chow + fruits/vegetables | 511-5  Individual cage, standard facility |
| 98064 | Oldest | female | 16.9 | 23 | | Clinically healthy | Standard macaque chow + fruits/vegetables | 511-6  Individual cage, standard facility |
| 98335 | oldest | male | 11.5 | 23 | | Clinically healthy | Standard macaque chow + fruits/vegetables | 511-7  Individual cage, standard facility |
| 98093 | oldest | male | 13.6 | 23 | | Clinically healthy | Standard macaque chow + fruits/vegetables | 511-8  Individual cage, standard facility |
| 00069 | oldest | male | 12.4 | 21 | | Clinically healthy | Standard macaque chow + fruits/vegetables | 511-9  Individual cage, standard facility |
| 095000 | old adult | female | 16.3 | 12 | | Clinically healthy | Standard macaque chow + fruits/vegetables | 511-10  Individual cage, standard facility |
| 095001 | Old adult | male | 15.2 | 12 | | Clinically healthy | Standard macaque chow + fruits/vegetables | 511-11  Individual cage, standard facility |
| 095002 | old adult | female | 14.9 | 12 | | Clinically healthy | Standard macaque chow + fruits/vegetables | 511-12  Individual cage, standard facility |
| 095003 | old adult | male | 15.6 | 12 | | Clinically healthy | Standard macaque chow + fruits/vegetables | 510-1  Individual cage, standard facility |
| 095004 | old adult | female | 17.2 | 12 | | Clinically healthy | Standard macaque chow + fruits/vegetables | 510-2  Individual cage, standard facility |
| 095010 | old adult | female | 16.5 | 12 | | Clinically healthy | Standard macaque chow + fruits/vegetables | 510-3  Individual cage, standard facility |
| 095018 | old adult | female | 13.3 | 12 | | Clinically healthy | Standard macaque chow + fruits/vegetables | 510-4  Individual cage, standard facility |
| 080960 | old adult | female | 12.4 | 13 | | Clinically healthy | Standard macaque chow + fruits/vegetables | 510-5  Individual cage, standard facility |
| 080810 | old adult | female | 16.9 | 13 | | Clinically healthy | Standard macaque chow + fruits/vegetables | 510-6  Individual cage, standard facility |
| 191013 | young | male | 9.4 | 7 | | Clinically healthy | Standard macaque chow + fruits/vegetables | 510-7  Individual cage, standard facility |
| 191003 | young | male | 8.3 | 7 | | Clinically healthy | Standard macaque chow + fruits/vegetables | 510-8  Individual cage, standard facility |
| 191000 | young | female | 10.6 | 7 | | Clinically healthy | Standard macaque chow + fruits/vegetables | 510-9  Individual cage, standard facility |
| 197007 | young | male | 11.5 | 7 | | Clinically healthy | Standard macaque chow + fruits/vegetables | 510-10  Individual cage, standard facility |
| 191001 | young | male | 8.7 | 7 | | Clinically healthy | Standard macaque chow + fruits/vegetables | 510-11  Individual cage, standard facility |
| 195041 | young | male | 10.2 | 7 | | Clinically healthy | Standard macaque chow + fruits/vegetables | 510-12  Individual cage, standard facility |
